# Using Graphene-Based Biosensors to Detect Dopamine for Efficient Parkinson’s Disease Diagnostics

**DOI:** 10.3390/bios11110433

**Published:** 2021-10-31

**Authors:** Małgorzata Kujawska, Sheetal K. Bhardwaj, Yogendra Kumar Mishra, Ajeet Kaushik

**Affiliations:** 1 Department of Toxicology, Poznan University of Medical Sciences, Dojazd 30, 60-631 Poznań, Poland; 2 Van’t Hoff Institute for Molecular Sciences, University of Amsterdam, Science Park 904, 1098 XH Amsterdam, The Netherlands; sheeturo@gmail.com; 3 Amsterdam Scientific Instruments B.V., Science Park 106, 1098 XG Amsterdam, The Netherlands; 4 Mads Clausen Institute, NanoSYD, University of Southern Denmark, Alison 2, 6400 Sønderborg, Denmark; mishra@mci.sdu.dk; 5 NanoBioTech Laboratory, Health System Engineering, Department of Environmental Engineering, Florida Polytechnic University, Lakeland, FL 33805, USA; ajeet.npl@gmail.com

**Keywords:** dopamine, Parkinson’s disease, graphene, point-of-care, biosensing

## Abstract

Parkinson’s disease (PD) is a neurodegenerative disease in which the neurotransmitter dopamine (DA) depletes due to the progressive loss of nigrostriatal neurons. Therefore, DA measurement might be a useful diagnostic tool for targeting the early stages of PD, as well as helping to optimize DA replacement therapy. Moreover, DA sensing appears to be a useful analytical tool in complex biological systems in PD studies. To support the feasibility of this concept, this mini-review explores the currently developed graphene-based biosensors dedicated to DA detection. We discuss various graphene modifications designed for high-performance DA sensing electrodes alongside their analytical performances and interference studies, which we listed based on their limit of detection in biological samples. Moreover, graphene-based biosensors for optical DA detection are also presented herein. Regarding clinical relevance, we explored the development trends of graphene-based electrochemical sensing of DA as they relate to point-of-care testing suitable for the site-of-location diagnostics needed for personalized PD management. In this field, the biosensors are developed into smartphone-connected systems for intelligent disease management. However, we highlighted that the focus should be on the clinical utility rather than analytical and technical performance.

## 1. Introduction

Parkinson’s disease (PD) is the second most common human neurodegenerative disorder, after Alzheimer’s disease (AD), with its incidence ranging from 10 to 18 per 100,000 people/year. Age is the most significant risk factor, with severe implications for public health. As populations are aging and life expectancy is rising worldwide, the number of people with PD is expected to increase by more than 50% by 2030 [1]. The disease is diagnosed based on motor impairment, including bradykinesia rigidity or tremor; this is when about 70% of the dopaminergic neurons of the substantia nigra pars compacta are degenerated due to α-synuclein deposits. PD is also diagnosed clinically once the synucleinopathy is already advanced. Researchers and clinicians indicate a potential temporal window before the onset of specific signs and symptoms of the disorder during which potential disease-modifying therapy could be administered to prevent or delay the disease development and progression. Indeed, there is a need for an early diagnosis primarily based on quantifiable measures (i.e., biomarkers) to refine qualitative assessments [2]. From a neurochemical perspective, PD is a neurodegenerative disease in which depletion of the catecholamine DA in the nigrostriatal system appears due to the loss of nigral neurons and striatal terminals. Over the years, the neurotransmitter loss progresses to reach only 3% of normal DA concentration in the putamen of patients with pathologically proven end-stage PD. In untreated PD patients, most studies found significantly decreased DA levels in the cerebrospinal fluid (CSF), reflecting dopaminergic cell loss [3]. Eventually, an individual develops motor symptoms, including bradykinesia, rigidity, tremor, and postural instability, which result from this drop in DA level. This means that DA level measurement might be a useful diagnostic tool for targeting the early stage of the defunctionalization of DA-producing neurons (nigrostriatal dopaminergic denervation) to enable the development of approaches to retard progression or even prevent the disease [4].

Dopamine replacement therapy (DRT), with levodopa as the gold standard drug treatment, is used to alleviate PD’s symptoms. While DRT does not cure the disease, it does help to reduce many of the motor symptoms of PD, especially during the first years after clinical disease onset. However, as the disease progresses, levodopa’s alleviating effect alters nonlinearly due to compensatory mechanisms for the depletion of the striatal DA level [5]. Véronneau-Veilleux et al. have reported that the compensation for denervation progress affects both levodopa’s duration and delayed effect [5]. They have highlighted that therapeutic doses of levodopa may have no effect at high levels of denervation, or that its effect may vanish rapidly, while larger doses of levodopa may cause high transient peaks in brain DA concentration, resulting in dyskinesias. The nonlinear pharmacodynamics of levodopa through PD progression complicates the optimization of a drug regimen. Indeed, as the disease progresses, side effects appear, and therefore personalized therapy is recommended. In light of recent findings, algorithmic approaches to dosing adjustments based on the measurement of the physiological and pharmacokinetic parameters by sensors are a promising step toward optimizing levodopa therapy [5,6].

Since DA is the target neurotransmitter both in PD diagnostics and treatment, the sensitive and selective methods of its determination have been of great interest for research and clinical implications. Notably, a low detection limit is essential due to very low DA concentrations in the body fluids, which is as low as 0.01–1 μM [7], including plasma up to 0.11 nM [8], CSF with levels amounting to 0.02–0.07 nM [8,9], and below the upper reference limit (3.3 μmol/24 h) in the urine of adults [10]. In the brain, the DA level is 83, 1130, and 2969 fmol/mg wet weight, in the cortex, putamen, and caudate regions, respectively, of PD patients [4]. During the last decade, numerous research efforts have been devoted to developing various techniques for DA quantification in body fluids, such as blood and CSF, including mass spectrometry coupled with separation techniques and immunochemical, fluorescence-based, and electrochemical methods [11]. Although these highly reliable approaches are generally well accepted, they still suffer from the disadvantages of being high cost, time consuming, and laborious, with requirements for highly skilled personnel [7].

**Figure 1:**
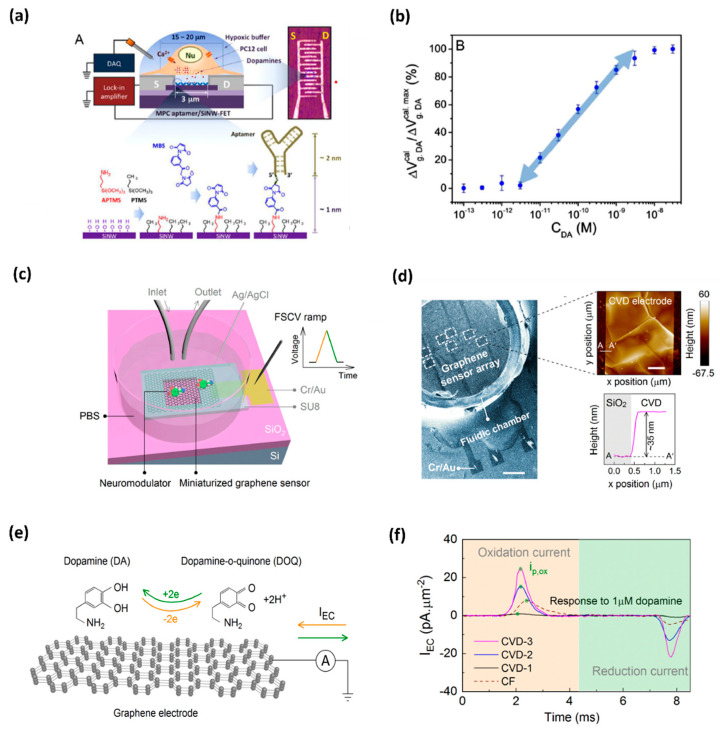
(**a**) DNA-aptamer-modified MPC SiNW-FET biosensor for dopamine; illustration of FET device for detecting exocytotic dopamine under hypoxic stimulation from living PC12 cells; (**b**) a semi-log plot of response as a function of dopamine concentration [17]. (**c**) Schematics of a graphene-based electrode used for measurements of DA; graphene electrode is mounted on a SiO_2_/Si substrate, and a fluidic chamber is filled with PBS solution containing target dopamine; (**d**) SEM image of the graphene-based sensor array; AFM topographic image of CVD grown multilayer graphene (**e**) mechanism behind the FSCV measurements of dopamine; and (**f**) noticeable area-normalized electrochemical current (I_EC_) response to the dopamine concentrations [18].

Due to high spatial and temporal resolution, high sensitivity and selectivity, and the possibility of direct monitoring at low cost and with the leverage of user-friendly tools, oxidation-based electrochemical sensing platforms are becoming a more popular and developed technique that is being implemented in a biological environment [12,13,14] and also for DA detection [15]. Efforts have been made to detect in situ DA, e.g., in the brain or living cells. Asif et al. applied the Zn-NiAl LDH/rGO superlattice electrode to track the DA released from human neuronal neuroblastoma cell line SH-SY-5Y [16]. Li et al. demonstrated a developed nanoelectronic biosensor, as shown in Figure 1, for monitoring the DA release from living PC12 cells [17]. Figure 1a shows the illustration of a DNA-aptamer modified by a multiple parallel-connected (MPC) silicon nanowire field-effect transistor (SiNW-FET) device, as well as the process of DNA-aptamer immobilization of the MPC SiNWFET. This device detects the DA under hypoxic stimulation from living PC12 cells. This developed MPC aptamer/SiNW-FET device demonstrated a DA detection limit of up to <10^−6^ M with high specificity when exposed to other chemicals, such as tyrosine, ascorbic acid (AA), phenethylamine, norepinephrine, epinephrine, and catechol. Wu et al. fabricated reproducible miniaturized, multi-layered, graphene-based sensors with astonishingly high sensitivity when compared with other sensors [18]. Figure 1b (i) shows the nanofabricated miniaturized multilayer graphene sensor electrodes. Figure 1b (ii) shows the scanning electron microscopy (SEM) image of the top of the sensor array and the AFM image of the sensor surface. Figure 1b (iii) depicts the mechanism behind it. The DA undergoes a redox reaction and is oxidized to dopamine-o-quinone (DOQ) by applying voltage. The sensitivity of the fabricated sensor is monitored by fast-scan cyclic voltammetry (FSCV) measurements. Figure 1b (iv) displays the area-normalized electrochemical current (I_EC_) curves in response to the DA solution. The fabricated graphene sensor achieved a high sensitivity of 177 pAμm^−2^μM^−1^ in response to the DA. It is concluded that the MPC aptamer/SiNW-FET sensor has shown improved specificity and an LOD up to <10^−11^ M for exocytotic DA detection, as compared to other existing electrochemical sensors. The real-time monitoring of DA induced by hypoxia demonstrates that for triggering the DA secretion, intracellular Ca^2+^ is required, which is commanded by extracellular Ca^2+^ influx instead of the release of intracellular Ca^2+^ stores. Such a device, capable of coalescing with living cell systems, opens a new gateway towards the biosensor for the futuristic studies of clinical disease diagnostics.

Moreover, downscaling the sensors enables limiting the sample volume, which is highly desirable for scarcely abundant specimens, including CSF or experimental research with small laboratory animals [11,19]. Therefore, DA sensing appears to be an applicable research use only (RUO) analytical tool for monitoring this biomarker in complex biological systems on studying PD, despite its high clinical relevance. However, graphene-based DA sensors are emerging analytical tools for PD diagnostics, as carefully and critically explained in this comprehensive review. Moreover, the challenges relating to the need for point-of-care (POC) testing is also discussed in this report. 

## 2. Analytical Performances of DA Graphene-Based Biosensors

Detecting biomolecules in real samples is associated with the interaction of other compounds with similar oxidation potentials during detection [20]. Thus, designing sensors for the DA monitoring in biological samples, such as routine clinical ones, is challenging since electrochemically active compounds commonly found in body fluids, such as AA, uric acid (UA), and glucose (Glu), constantly interact with each other during detection due to their similar oxidation potentials. Moreover, the present macromolecules, including proteins, can non-specifically adsorb on the electrode surface, thus hindering the electron transfer rate [21]. Thus, the development of electrochemical methods for the analysis of DA in a complex matrix must address all these possible interactions to enable its successful DA detection in a simple, rapid, and highly selective way.

**Figure 2:**
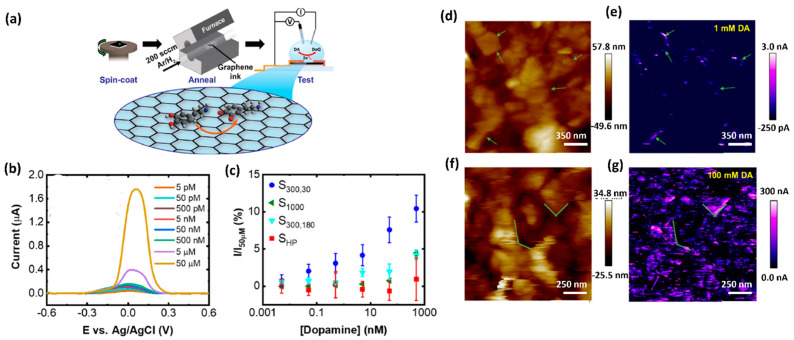
(**a**) Schematic representation of fabrication and electrochemical testing process of the graphene ink-based DA sensor. (**b**) Differential pulse voltammogram of the response towards DA detection from 5 pM to 50 μM. (**c**) Normalized peak current values versus DA concentration. (**d**) Height map, measured using scanning electrochemical microscopy (SECM) and (**e**) the corresponding electrochemical map with 1 mMDA. (**f**) A height map of a different region of the graphene film and (**g**) the corresponding electrochemical map with 100 mM DA [22].

The limitation caused by overlapping voltametric signals of compounds with very close oxidation potentials and relatively poor selectivity can be avoided by applying different sensing layers that enable separate detection of the electrochemical signals. Several electrode-modification substances, such as oxides, conducting polymers, and nanomaterial, have been adopted for this purpose. Nanomaterial-modified electrodes, especially with graphene and its derivatives, such as reduced graphene oxide (rGO) and graphene oxide (GO), have recently attracted great focus in electrochemical biosensing approaches [7,20,22,23,24,25,26]. Due to their unique structure, graphene-based materials increase the conductivity of the compounds used in electrochemical measurement systems. Owing to their large surface area, they offer a high number of accessible active sites to detect analytes (Figure 2) [24]. Graphene is always admired for its excellent properties among the various sensing materials for DA due to its excellent electrical conductivity and π−π interaction between the aromatic rings of DA and graphene. Butler et al. developed a graphene ink-based, ultrasensitive electrochemical sensor for the detection of DA. The lowest limit of detection is reported as 1 nM. This sensitivity and selectivity of the sensor are achieved by tuning the surface chemistry of graphene. Figure 2a shows a schematic illustration of the fabrication of the DA sensor. The curves of Figure 2b depict the effect of annealing the graphene towards the DA response from 55 pM to 50 μM, using DPV measurements. Scanning electrochemical microscopy (SECM) mapping confirmed that the graphene layer (Figure 2d−g) shows higher oxidation at the edges of the flakes. Figure 2d,f display the height maps for two different regions of the graphene ink film-based sensor. Figure 2, for example, shows the electrochemical mapping of the graphene ink with 100 mM DA in PBS. At different concentrations, the total activity is enhanced, as seen by the increased magnitude of the current in the electrochemical response. Considering the 2D defects and the active edge sites of graphene ink, it can be an ideal candidate for printable and low-cost DA sensing devices/systems.

Butler et al. developed ultrasensitive graphene ink which enabled facile post-deposition annealing of electrochemical sensor for DA detection with the lowest detection limit of 1 nM [21,22]. Furthermore, by increasing the affinity of the cationic DA form to the materials’ surface, electroactive oxygen groups in graphene materials play a significant role in its detection [27]. Graphene can also be easily modified with various nanomaterials to attain an enhanced catalytic effect [21]. However, the abovementioned advantages of graphene are limited due to the strong π–π stacking and van der Waals interactions. Therefore, surface modifications of the graphene nanosheets, made to improve its functionalization, must, to be effective, reduce these unfavorable effects while also providing enhancement of the electrocatalysis of graphene, increasing the surface area, and improving the conductivity of the composite materials. Moreover, the biofunctionalization aims not only to improve the analytical performance characteristics, such as sensitivity and selectivity, but also to enable miniaturization of the diagnostic platform to make it convenient for the analysis of real and complex matrices, and to make it able to perform monitoring in real time, as well as in in vivo testing [21]. 

**Figure 3:**
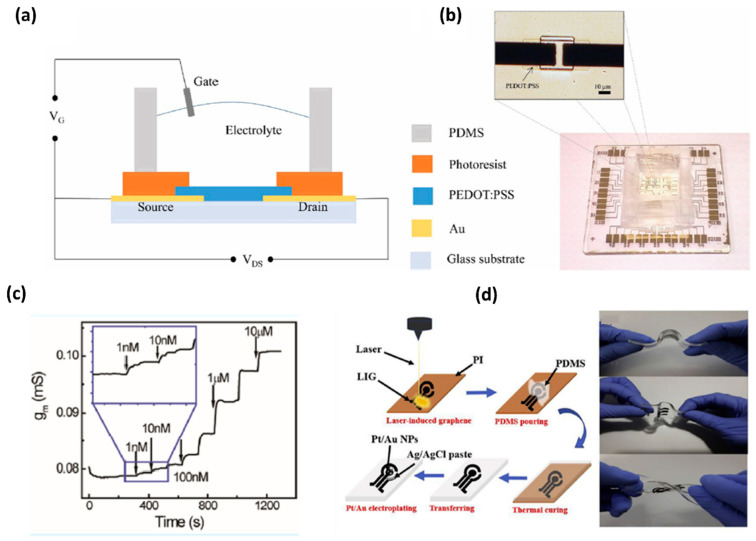
(**a**) Schematic diagram of an OECT device for DA sensing. (**b**) Optical image of the transistor and the whole OECT array. (**c**) Channel transconductance (gm) response to additions of DA with different concentrations [28]. (**d**) Fabrication of flexible electrochemical DA sensor with a Pt-AuNPs/LIG/PDMS electrode and display of flexibility of the fabricated electrode [29]. Table 1 summarizes the analytical performances of DA biosensors as claimed by various reports [9,30,31,32,33,34,35,36,37,38,39,40,41,42,43,44,45,46,47,48,49,50,51,52,53,54,55,56,57,58,59,60,61].

Wang et al. developed organic electrochemical transistors (OECT) for accurate sensing of DA based on the alternative current (AC) measurements [26], as shown in Figure 3. This advanced method was introduced to characterize the behavior of ionic motion and the ion concentrations in aqueous electrolytes, as well as the rapid electrochemical detection of DA with an LOD of 1 nM. This AC method gives a stable and accurate signal in a broad frequency range and a low noise level by introducing a lock-in amplifier. Therefore, the AC method opened a new window for OECT-based sensors [28]. Xue-Xui et al. developed a high-flexibility and high-selectivity DA sensor with a simple fabrication process. Thus, the fabricated Pt–Au/LIG/PDMS sensor exhibited a sensitivity of 865.8μA/mM cm^−2^ and a limit of detection of 75 nM, and successfully detected DA in human urine. The flexibility of the sensor offers the possibility for continuous DA monitoring in future self-care monitoring systems [29]. In Table 1, we have presented various graphene modifications developed in electrodes for DA detection, along with their analytical performances and interference studies, which are listed based on their limit of detection (LOD) in different types of biological samples.

**Figure 4:**
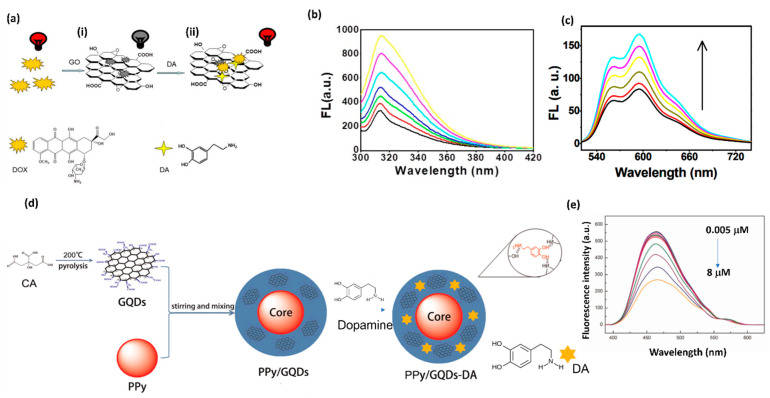
(**a**) Schematic illustration of DOX-GO complex and its fluorescence response along with the (**i**) turn off and (**ii**) turn on mechanism towards DA detection. (**b**) Fluorescence spectra of DOX-GO-DA solutions with the addition of DA concentrations ranging from 1.5 μM to 6.0 μM with excitation at 280 nm. (**c**) Fluorescence emission spectra of the DOX-GO for DA detection in human serum at fluorescence intensity of 598 nm. (**d**) Design of PPy/GQDs. (**e**) Fluorescence emission of spectra of PPy/GQDs with increasing concentrations of DA from 0.005 to 8 μM.

Along with the electrochemical biosensors, fluorescence biosensors are attractive due to their high sensitivity and rapid response. In terms of signal transduction, fluorescence biosensors are categorized as fluorescence resonance energy transfer (FRET) [62], chemiluminescence [63], fluorescence dye staining [64], fluorescent probe [65], and fluorescence anisotropy [66] biosensors, and have been proven to be promising devices for diagnostics. The GO derivatives of graphene have the ability to quench the fluorescence of the adsorbed dyes due to their conjugated structure. A. Teniou et al. developed GO-based fluorescent aptasensor for DA detection [62]. In this sensor, there is a fluorescence resonance energy transfer (FRET) device where GO plays the role of an energy donor and a carboxyfluorescein (FAM)-labeled aptamer is the energy acceptor. The thus-developed GO-based aptasensor depicts a linear relationship between DA concentration (3 to 1680 nm) and fluorescence recovery. The calculated value of the LOD is 0.031 nM. R. Cheng et al. developed a label-free doxorubicin (DOX)-GO fluorescence sensor for DA detection in cells and the human serum (Figure 4a) [63]. DA has strong adsorption towards the GO as compared to the DOX. The exposure of DA to the DOX-GO leads to the release of pre-absorbed DOX from the same DOX-GO platform, leading to the recovery of the quenched fluorescence (DOX). This quenching turns on the sensor. The DOX-GO platform shows a linear range from 8.3 × 10^−7^ M to 3.3 × 10^−5^ M in aqueous solution (curves in Figure 4b) and 1.44 to 11.48 μmol L^−1^ in human serum (curves in Figure 4c) for DA detection. Therefore, the DOX-GO label-free sensor successfully detected DA in the living cells. Another research group, Zhou and coworkers, tested DA concentrations with the polypyrrole/graphene quantum dots core/shell (Ppy/GQD) hybrids sensor, as shown in Figure 4d [67]. The Ppy/GQD exhibits strong fluorescence emission. The prepared sensor shows a decrease in the fluorescence intensity along with the increasing concentration of DA and shows a linear range from 5–8000 nM (Figure 4e) with an LOD of 10 pM (S/N = 3). Thus, the developed sensor can easily detect DA when exposed to real human blood samples. The fluorescence approach is the state of the art for developing low-cost, simple, and sensitive sensors for DA detection in living cells. 

## 3. Challenges and Perspectives towards POC Diagnostics of DA

The detection of DA has been of great interest for clinical implications because the neurotransmitter can be used as a biomarker for PD diagnosis, and which can help with monitoring the disease progression and its treatment effectiveness [68]. In fact, as the disease progresses and side effects appear, individualization of therapy is recommended. Because of the nonlinearities of levodopa, DA, and basal ganglia dynamics, which account for PD progression, there is an unmet need to estimate individuals’ parameters, including DA level, for DRT dosing adaptation. So far, algorithms have been developed to tailor DRT based on information acquired by wearable sensors which estimate the physiological and pharmacokinetic parameters [5,6]. Simultaneous monitoring of DA levels could improve individualized drug regimen optimization and help predict sudden waning in levodopa’s effect. The development of in vivo sensing devices is currently in its beginning; the currently available electrochemical devices dedicated to DA detection are too large for on-field inspection [21]. 

Fulfilling this goal is associated with moving away from time- and cost-consuming laboratory analysis that requires skilled technicians to point of care testing (POCT), i.e., medical tests performed close to the site of patient care. The POC devices face significant challenges for achieving reliable results quickly (a few minutes) without sample pretreatment. They should be portable and user-friendly while providing acceptable analytical performance and clinical significance. Electrochemical sensors meet the main requirements of POCT, such as sensitivity, selectivity, ease of handling, affordability, disposability, stability, and flexibility. Electrochemical biosensors, which can be miniaturized, facilitate work with real samples in small volumes (μL-nL) without any pretreatment and versatility due to multiple sensor arrays, and show advantages compared to optical biosensors when used in POC devices [69].

**Figure 5:**
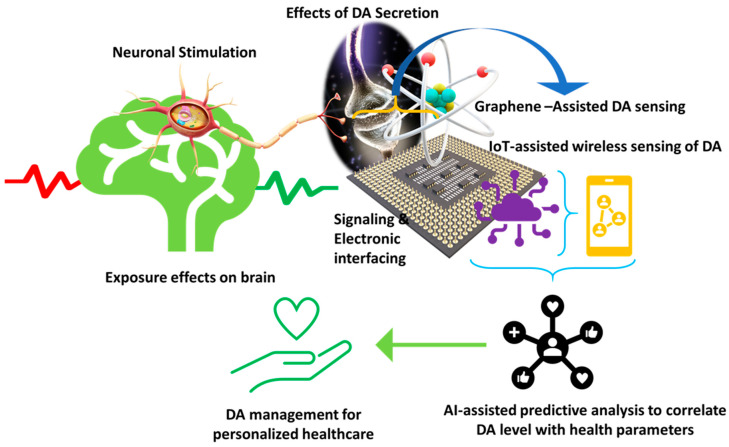
Illustration of a futuristic approach based on sensor-IoT-AI-goal of PD management.

Considering the acceptable selectivity and sensitivity of the graphene-modified electrochemical biosensors for DA as depicted in Table 1, and the simplicity of the measurement process, they can potentially be applied to POC testing [70]. Hence, developing a portable and miniaturized sensing platform for DA detection is significant for this approach. Moreover, since electrochemical biosensors can be easily combined with digital signal readout, smartphone-based integrated systems for simultaneous detection of biomolecules, including DA, have been developed (Figure 5). They allow real onsite measurement of DA, which can immediately be shared with the clinician [69]. The systems usually consist of a disposable sensor with a graphene-modified electrode, a coin-size detector, and a smartphone equipped with application software. Ji et al. demonstrated linear, high sensitivity, and specific detection of the electrochemical activity of biomolecules, including DA, in biological matrices with the use of the smartphone-based integrated system, supporting its use for DA detection in POC testing [20]. Recently, Yu et al. have reported achieving a turn-on visual DA assay-based ratio metric fluorescence paper microchip coupled with a smartphone-assisted portable detection device for POC testing (POCT) [62]. Moreover, The role of the rapid improvement of smartphone cameras in optical POC sensing should also be considered [69]. This supports the DA detection strategy trend based on the use of smartphones for portable, rapid, and accurate POCT [71,72]. 

Another area of research that still requires increased attention is the development method for noninvasive DA detection with acceptable reproducibility and stability in clinical diagnostics. In this sense, the measurement of salivary DA without pretreatment or modification of the samples, and with satisfactory results that are comparable to the clinical test, is highly desirable. First, however, it should be highlighted that the DA level in human saliva is ca. <0.5 nM [68,73].

The POCT approach appears to be a promising step toward optimizing DRT and clinical trial designing as well; however, it requires translation of the findings into a mobile health decision tool. As Lingervelder et al. have reviewed, for general practitioners, the clinical utility of POC testing is the most critical aspect [65]. To ensure POCT’s usefulness to clinicians, future research [74], despite focusing on the analytical and technical performances of a test, should also tackle the aspects relating to the clinical utility and risks [75,76,77,78,79,80,81,82,83]. Moreover, in the case of smartphone-connected POCT devices, the issues related to data sensitivity, including privacy and protection against theft and medical advice, should be addressed [69].

## 4. Conclusions and Viewpoint

The graphene-based biosensors offer promising diagnostic potential for DA detection, with acceptable selectivity and sensitivity in human serum/plasma and urine samples with an LOD ranging from 1 pM to 1.5 µM. Notably, the research presented herein meets the LOD of salivary DA level. Considering new perspectives of the development of portable and miniaturized sensing platforms, which can be improved through integration into smartphone-based systems, graphene biosensors appear to be serious candidates for such application in DA sensing. However, to ensure the POCTs’ usefulness in PD diagnostics and to make the treatment more personalized and efficient, further development should not only focus on the analytical and technical performance aspects of a test, but also deal with the clinical utility and risks. Moreover, due to admirable sensing performances, including multichannel detection, high sensitivity, and fast response, graphene-based biosensing, despite the clinical relevance, appears to be a useful RUO tool for real-time detection of DA various biological systems and in animal experiments in PD research.

## Figures and Tables

**Table 1:** Analytical performances of DA biosensors.

Graphene Functionalization	LOD (μM)	Biological Samples	Interference Compound	Reference
3D RGO-PU	1.0 × 10^−6^	(h) urine, serum	AA, UA, Glu, CA, 4-NP, Trp, Tyr, GSH	[30]
PFSG/GCE	0.0008	(h) serum	AA, UA	[31]
ZnO NWAs/GF	0.001	(PD) serum	AA, UA	[32]
NiAl LDH/G LBL	0.002	SH-SY 5Y cells	AA, UA	[33]
Au NPs-CNT-G-pMet-SPCE	0.0029	(h) urine	UA	[7]
AgNCs/AgNPs/GO	0.00353	brain homogenate of PD mice	GSH	[34]
GR/GLN	0.0045	(h) urine, serum	AA, UA, Glu	[35]
Fe_3_O_4_/rGO/GCE	0.005	(h) urine	UA, CA, Glu, AA, NaCl, AP	[36]
Fe_3_O_4_@GNs/Nafion/GCE	0.00713	(h) urine, plasma	AA, UA	[37]
graphene-MoS_2_/GCE	0.007	(b) serum	AA, UA, CA, Glu, cysteine, Na^+^, K^+^, Mg^2+^, Ca^2+^, Cl^−^	[38]
Fe_3_O_4_-SnO_2_-G/CPE	0.0071	(h) urine, serum	AA, UA	[39]
RGO/Mn-TPP/GCE	0.008	(h) serum	AA, UA	[40]
Ag NPs/GO/P(Arg)/GCE	0.01	(h) urine	U, CA, Glu, Na^+^, K^+^, L-lysine, L-cysteine	[41]
TiN-RGO/GCE	0.012	(h) urine	AA, UA, Glu, LA	[42]
PA/GO/GCE	0.016	(h) urine	AA, UA	[43]
GNCs/CMG/GCE	0.02	(h) serum	AA	[44]
Au–Pt/GO–ERGO	0.0207	(h) serum	AA, 5-HT, UA, AP, EP, NEP, CA, Glu, H_2_O_2_, NaCl, KCl, KNO_3_, Na_2_SO_4_, ZnCl_2_, CaCl_2_, (b) serum albumin, immunoglobulin	[45]
α-Fe_2_O_3_@erGO/GCE	0.024	(h) serum	AA, UA, Glu, U, H_2_O_2_, NaCl, KCl	[46]
CNDs-RGO/GCE	0.03	(h) serum	UA	[47]
Au-ZnO NCAs/GF	0.04	(h) urine	UA	[48]
Pt/rGO/MEA	0.05	(r) CPU	AA, UA, Glu, U, 5-HT, DOPAC	[49]
rGO–Cu_2_O/GCE	0.05	(h) urine, blood	AA, UA	[50]
PANI/Fe_2_O_3_-SnO_2_/rGO/PFSG/GCE	0.076	(a) urine	UA	[27]
PTPCNs/GCE	0.078	DA injection and urine	UA	[51]
ERGO/PLL/GCE	0.10	(h) urine	AA, UA	[52]
3D-NG	0.26	(h) urine	AA, UA, AP	[53]
GO/Au NPs	0.29	(a) urine	UA, AA	[20]
AG-NA/GCE	0.33	(h) urine	AP	[54]
GO-BAMB-Co(OH)_2_	0.4	(h) urine	AA, 5-HT	[55]
Pd-GR/nano-CILE	0.5	(h) urine, serum	UA	[56]
3D HGB/ITO	1.0 *	(h) plasma	UA	[57]
Pdop@GR/MWCNTs	1.0	(h) urine, serum	AA, UA	[58]
RGO–ZnO/GCE	1.08	(h) urine, plasma	AA, UA	[59]
Au/RGO/GCE	1.4	(r) serum	AA, UA, CA, NaCl, KCl, NaNO3, CaCl_2_, Glu, cysteine	[60]
mp-GR/GCE	1.5	(h) serum	UA	[61]

* levodopa: 3D HGB/ITO—3-dimentional hollow graphene balls using nickel nanoparticles/the indium tin oxide glass electrode; 3D-NG—three-dimensional nitrogen-doped graphene; 3D RGO-PU—3D-reduced graphene oxide/polyurethane; 4-NP—4-nitrophenol; 5-HT—serotonin; α-Fe_2_O_3_@erGO—magnetic hematite-decorated electrochemically reduced graphene oxide; (a)—artificial; AA—ascorbic acid; AG-NA—activated graphene-Nafion; AgNCs/AgNPs/GO—Ag44(SR)30 nanoclusters (AgNCs) with 5-mercapto-2-nitrobenzoic acid (MNBA)/silver nanoparticles/graphene oxide; Ag NPs/GO/P(Arg)—silver nanoparticles/graphene oxide/poly(L-arginine); AP—acetaminophen; Au NPs-CNT-G-pMet—gold nanoparticles-carbon nanotube-graphene-poly(L-methionine); Au–Pt/GO–ERGO—Au–Pt bimetallic nano-clusters/graphene oxide electrochemically reduced; Au/RGO—gold nanoplates/reduced graphene oxide; Au-ZnO NCAs—gold nanoparticles-Zinc oxide nanocone arrays; (b)—bovine; CNDs-rGO—carbon nitride dots-reduced graphene oxide nanocomposites; CA—citric acid; CPU—the caudate putamen; DOPAC—3,4-dihydroxyphenylacetic acid; EP—epinephrine; ERGO/PLL—electrodeposited reduced graphene oxide/polymerization of L-lysine; Fe3O4@GNs/Nafion—Nafion covered core–shell structured Fe_3_O_4_@graphene nanospheres; Fe_3_O_4_/rGO—iron oxide/graphene oxide; Fe_3_O_4_-SnO^2^-Gr/CPE —iron oxide/tin oxide/carbon paste electrode; GCE—glassy carbon electrode; GF—graphene foam electrode; Glu—glucose; GNCs/CMG—gold nanocages/chemically modified graphene oxide; GO-BAMB-Co(OH)_2_—graphene oxide -1,4-bis(aminomethyl)benzene and cobalt hydroxide; GONRs—graphene oxide nanoribbons; graphene-MoS_2_—graphene and molybdenum disulfide hybrids; GR/GLN—graphite sheets assisted with gelatine; GSH—reduced glutathione; (h)—human; LA—lactic acid; LOD—limit of detection; mp-GR—multi-nanopore graphene; NEP—norepinephrine; NiAl LDH/G LBL—positively charged NiAl layered double hydroxides nanosheets/negatively charged monolayers of graphene layer by layer; PANI—polyaniline; PA/GO—phytic acid/graphene oxide; PD—Parkinson’s disease patients; Pd-GR/nano-CILE—palladium-doped graphene/nano-carbon ionic liquid electrode; Pdop@GR/MWCNTs—polydopamine/graphene/multiwalled carbon nanotubes; PFSG—poly(sodium 4-styrenesulfonate)-functionalized three-dimensional graphene; Pt/rGO MEA—platinum nanoparticles and reduced graphene oxide/microelectrode array; PTPCNs—porous tal palm carbon nanosheet; (r)—rat; rGO–Cu_2_O—copper (I) oxide nanostructure decorated reduced graphene oxide; RGO/Mn-TPP—reduced graphene oxide/manganese tetraphenylporphyrin; RGO–ZnO—reduced graphene oxide-zinc oxide; SPCE—screen-printed carbon electrode; TiN-RGO—reduced graphene oxide and titanium nitride, Trp—Tryptophan; Tyr—Tyrosine; U—urea; UA—uric acid; ZnO NWA—ZnO nanowire arrays.

## Data Availability

The datasets analyzed during the current study are available from the corresponding author on reasonable request.
